# Global evolutionary isolation measures can capture key local conservation species in Nearctic and Neotropical bird communities

**DOI:** 10.1098/rstb.2014.0013

**Published:** 2015-02-19

**Authors:** David W. Redding, Arne O. Mooers, Çağan H. Şekercioğlu, Ben Collen

**Affiliations:** 1 Centre for Biodiversity and Environment Research, University College London, Gower Street, London WC1E 6BT, UK; 2 Department of Biological Sciences, Simon Fraser University, Burnaby, British Columbia, Canada; 3 Department of Biology, University of Utah, Salt Lake City, UT 84112, USA

**Keywords:** biogeography, community, extinction risk, phylogenetically distinct, phylogeny

## Abstract

Understanding how to prioritize among the most deserving imperilled species has been a focus of biodiversity science for the past three decades. Though global metrics that integrate evolutionary history and likelihood of loss have been successfully implemented, conservation is typically carried out at sub-global scales on communities of species rather than among members of complete taxonomic assemblages. Whether and how global measures map to a local scale has received little scrutiny. At a local scale, conservation-relevant assemblages of species are likely to be made up of relatively few species spread across a large phylogenetic tree, and as a consequence there are potentially relatively large amounts of evolutionary history at stake. We ask to what extent global metrics of evolutionary history are useful for conservation priority setting at the community level by evaluating the extent to which three global measures of evolutionary isolation (evolutionary distinctiveness (ED), average pairwise distance (APD) and the pendant edge or unique phylogenetic diversity (PD) contribution) capture community-level phylogenetic and trait diversity for a large sample of Neotropical and Nearctic bird communities. We find that prioritizing the most ED species globally safeguards more than twice the total PD of local communities on average, but that this does not translate into increased local trait diversity. By contrast, global APD is strongly related to the APD of those same species at the community level, and prioritizing these species also safeguards local PD and trait diversity. The next step for biologists is to understand the variation in the concordance of global and local level scores and what this means for conservation priorities: we need more directed research on the use of different measures of evolutionary isolation to determine which might best capture desirable aspects of biodiversity.

## 1. Introduction

Making informed decisions about the appropriate focus of conservation investment has become a central theme of both academic research and conservation action. The discipline has been driven by both academia and conservation by the often-cited ‘agony of choice’—in essence, how to make the best decision with limited resources [1]. A variety of methods have been proposed for prioritizing those species most deserving of conservation attention; one candidate that is gaining traction prioritizes those species that maximize phylogenetic diversity (PD), or the sum of the edge lengths of the phylogenetic tree linking species [2]. Because time and divergence are likely to be correlated, species representing a greater proportion of independent evolutionary time on the tree represent more total evolution and so should be prioritized. Therefore, using metrics to assess and prioritize PD attempts to account for the fact that species differ substantially in the amount of complementary genetic information that they embody [3–5]. Such techniques are a practical way of accounting for the unequal contribution of species of conservation concern to biodiversity conservation. Building on this recognition, species-level measures that integrate evolutionary history and likelihood of loss of a given species have been developed over the past two decades [2,5–7]. The field of research has moved rapidly, with the consequent translation of theory [1,4,8–10] into conservation practice [6,11,12].

Despite this welcome development of global-scale metrics for species prioritization, the majority of conservation action is taken not under global level priority setting schemes, but at a local scale, typically at the level of ecological communities and populations. Understanding change in population and community-level metrics of diversity over time is critical to understanding change in biodiversity. The loss of populations from communities is a prelude to species extinction [13], and local reductions in taxonomic, genetic and functional diversity impact many different aspects of biodiversity. Whether and how global-scale metrics based on evolutionary history map to a local scale has received far less scrutiny to date than the development and implementation of global prioritization methods. The scale of distinction could be important. At a local scale, conservation-relevant assemblages of species are likely to be made up of relatively few species, possibly spread across a large section of the phylogenetic tree. This phylogenetic spread means that one or a few species may contribute a great deal to local PD, making the choice of priority setting algorithm important.

A local perspective on conservation phylogenetics intersects with a related field as well: as phylogenetic relationships among species has rapidly become easier and more reliable, community ecologists have become increasing interested in the evolutionary relationships among coexisting species for evaluating clustering, community assembly, functional differences and for mechanistic insights into community structure [14]. Importantly, community ecologists have also presented provocative evidence that the total PD in an assemblage is a good predictor of ecosystem function [15].

In this article, we seek to take a step towards integrating community-level phylogenetics with global conservation approaches for prioritizing evolutionary history. We ask to what extent global species-centred metrics of evolutionary isolation [6] are useful for conservation priority setting at the community level, specifically asking whether species that score highly for global metrics also score high when the metrics are measured on the tree spanning the species in a community assemblage and whether these species also contribute substantially to the total PD and trait diversity of the local community. We use a rich dataset from communities of Nearctic and Neotropical breeding birds to test the following:

1. —how do global and local metrics of three measures of evolutionary isolation (evolutionary distinctiveness (ED); unique PD contribution (PE) and average pairwise distance (APD)) relate to one another?
2. —does prioritizing globally high ED scoring species within a community select species with unusual sets of ecological traits at the local level? and
3. —is more local PD and trait variation lost when removing species from a community that score high in global evolutionary isolation compared with random species removal?

## 2. Material and methods

### (a) Community data

We organized published lists of species in bird communities across the Nearctic and Neotropics. We drew species identity from two vetted long-term datasets: the breeding bird survey (United States) and Christmas bird counts (North and South America). Species lists from all years for the same survey route were collapsed so that all species seen during all years' surveys were taken to represent the local ‘community’ along that route. To account for under-sampling, we removed those sites where surveys had recorded fewer than 25 species. We analysed a final dataset of 4628 communities of Nearctic and Neotropical birds containing 2662 species in total (median community size = 94 species, range 26–534).

Phylogenetic and trait data were derived from previously published sources. We downloaded 10 000 full species-level phylogenies from [12,16] and compiled trait data from [17] for all surveyed species. We chose ecological traits based on their suitability for explaining ecological separation, following [18]. Traits included body mass, primary habitat, habitat breadth, vertical distribution (ground to aerial), vertical breadth (range between seven levels from ground to aerial), diet (for each species, relative amount of food types consumed add up to 10), diet breadth, guild, social structure, nest type (14 types), nest substrate (11 types), mean clutch size and activity pattern. All quantitative data were standardized by their respective mean. We recognize that these are relatively few ecological variables from a large possible set; however, we do not know how much extra (orthogonal) information would be added by including more than this ecologically significant subset.

We matched species among the community, phylogenetic and trait datasets using Avibase as a reference [19]. Those species that were found in the community data but not in the phylogeny were dropped; in the case of sub-species, we simply substituted the parent species in the phylogeny.

### (b) Analyses

We calculated the median of each of three measures of evolutionary isolation across the 10 000 phylogenies, each at three scales: the length of the terminal branch [20,21] linking a species to the tree (unique PD contribution, also called phylogenetic endemism or pendant edge (PE) cophenetic, R package ape [22]); the APD, i.e. the mean APD to all other species in the tree (cophenetic, R package ape [22]) and the fair proportion measure of ED (ed.calc, R package caper [23]). These metrics include the two ends of an axis of evolutionary isolation measures that weights information nearer the root (APD) or nearer the tip (PE [24]), alongside the only currently used measure in active conservation prioritization (ED: [6,11]). For each species in each community, we calculated two ‘global’ scores, one based on the entire tree of the birds and another set of scores based on a continental level tree consisting of all the species in our dataset. Differences between the two sets of ED and PE scores were minimal (Spearman's rank correlation *ρ* = 0.975–0.999) and, given that we had only trait data for all species in our North American communities, hereon we used the scores calculated using the continental tree; for brevity, we refer to these as ‘global’ scores. Then, for comparison, we calculated the same three measures of isolation for each community using just the sub-tree linking the species in that community (‘local’ scores).

We calculated trait-based metrics based on distances between all species in the surveyed communities using the Gower approach (daisy R package cluster [25]). Any species pairs that had no data to compare were awarded the median distance for all other species. Minimum distance to any other species in the global tree (trait uniqueness, TU) and mean APD to all other species in the global tree (trait APD (TAPD)) were then calculated directly from the resulting distance matrix. The same approach as above was then applied to each local community separately, resulting in local values for these two measures.

We then produced datasets with and without species known to be alien invasives [26] and recalculated all local scores. To investigate the role of abundance, we also produced a dataset after removing all species that accounted for less than 1% of all individuals seen during the course of all surveys at the location, again recalculating local scores. Using all three datasets (full, no invasives, rare species removed), we calculated a set of rank correlation (Spearman's *ρ*; cor, R package base [27]) for each community, between global scores (APD, ED and PE) and all versions of local scores (i.e. ‘local’ APD, ED, PE, TU and TAPD).

To visualize the variation in the correlation between local and global scores across communities, we mapped the Spearman's *ρ* values onto the survey point locations in our study area. The observed pattern was spatially non-random (figure 2). To further explore this variation among communities, we chose one of the scores, ED, and modelled the correlation between local ED and global scores using linear mixed-effects models that account for the spatial autocorrelation between the survey points and the variables: years surveyed, latitude, species richness (total species seen), PD/species richness (total community PD divided by total species seen, as a measure of dispersal across the tree), distance to coast (measured as kilometres from Global Self-consistent Hierarchical High-resolution Geography coastlines http://www.ngdc.noaa.gov/mgg/shorelines/gshhs.html using R spDist function) and habitat class (data taken from Globcover 2009 calculated using R function *over*). We chose ED as it is the only measure of evolutionary isolation so far (to our knowledge) used actively in global conservation [6,11]. Diagnostic analyses (not reported) confirmed a spatial influence and by trial and error a model using spherical distribution had the best Akaike information criteria (AIC) score. AIC scores for the full model were calculated and terms removed manually, starting with the least correlated variable until AIC did not improve.

Finally, we ran a simulation to test the effect of using global conservation priority setting on capturing biodiversity at the community level. We first ranked all species by their three respective global evolutionary isolation scores. We selected the top scoring 500 species for each global metric and determined how many of these top-ranking species were in each community. We then removed these species from each community they were found in and measured biodiversity change in terms of mean reduction in local PD and change to mean average trait pairwise difference (measured using TAPD). The latter value measures how closely related species in a group are on average, with a reduction in this value occurring if species with more unusual traits being removed, i.e. the remainder is then more similar. For a comparison, we then removed the same number of species randomly from each community (replicated 500 times), to create an expectation of PD lost and TAPD change given random loss of species from communities.

## 3. Results

### (a) Phylogenetic diversity

**Figure 1.**
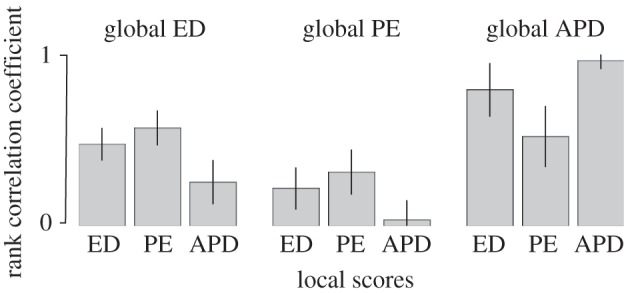
Mean correlation between three global evolutionary isolation scores (ED, PE and APD—definitions in text) and local scores measured at the community level for 4628 bird communities across North and tropical South America.

The correlations between global ED and local ED scores among the 4628 communities of birds were moderate (mean *ρ* across communities = 0.52, s.d. = 0.09). Correlations were marginally higher for global ED versus local PE (mean *ρ* = 0.59; s.d. = 0.09). There was a weaker relationship between global PE and local PE (mean *ρ* = 0.31, s.d. = 0.13). Surprisingly, the global APD was very closely related to local APD (figure 1; mean *ρ* = 0.96, s.d. = 0.04, 98% of communities significant at the 0.05 level) and global APD also covaried strongly with local ED (mean *ρ* = 0.79, s.d. = 0.15) and to a lesser extent with local PE (mean *ρ* = 0.50, s.d. = 0.17), though these latter results had relatively higher variance.

On the whole, the removal of alien species from the communities had a negligible effect on the correlations between global ED and local ED. Removing rarely seen (vagrant) species also had a limited but slightly positive effect on the majority of correlations.

**Figure 2.**
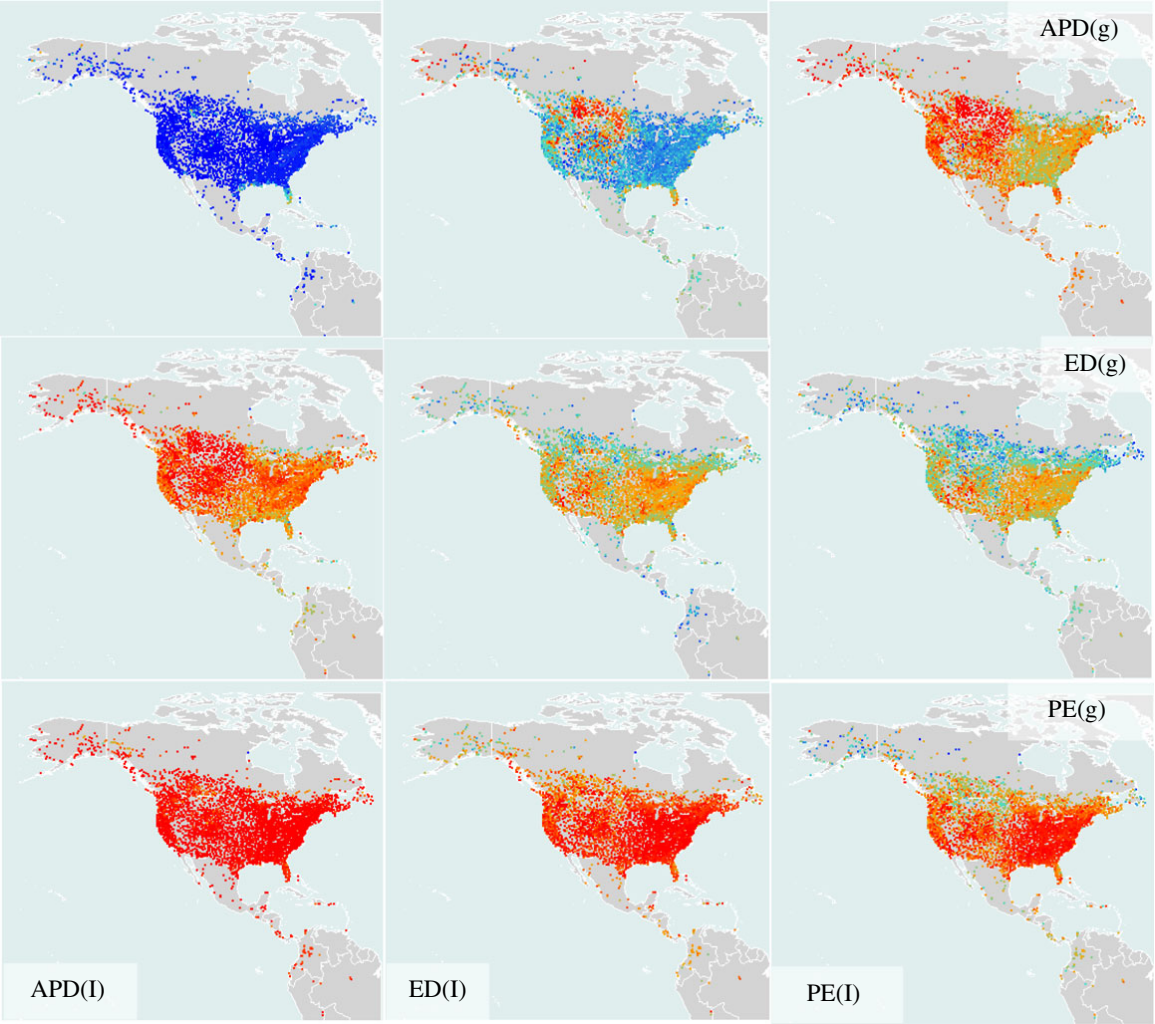
Map of correlations between local evolutionary isolation scores (l) and global evolutionary isolation scores (g) for 4628 bird communities for three different measures of evolutionary isolation—descriptions in text. Blue colours are higher correlation (light blue is a coefficient of approximately 0.7; dark blue represents a value tending to 1), orange-red colours show weaker correlation (red represents a correlation coefficient of 0 to around 0.4; orange around 0.55). (Online version in colour.)

The strength of the correlation between global ED and local ED was spatially non-random (figure 2), appearing to be strongest in the western Nearctic, and in central areas and towards northern limits of where bird communities were surveyed. There were especially strong relationships for communities in the central prairie regions of the United States as well as the northern boreal forest communities, and communities from the tropical wet forests in parts of South America, particularly Brazil (though for the latter, numbers of communities were low). Conversely, global APD showed weaker associations with local ED and local PE in the central prairie regions, and stronger correlations in eastern forest communities (figure 2).

These biogeographic patterns were apparent even when accounting for other factors, such as species richness and survey effort (table 1). Areas near the coast, and in forested habitats and grasslands had higher correlations between local and global ED scores, though the lack of differentiation in the land cover data into forest types, e.g. eastern (mainly deciduous) forests and boreal (mainly coniferous) forest, prevented more detailed comparisons (table 1).

**Table 1.** Regression coefficients from a linear mixed-effects model of the correlation coefficient between global ED scores and local ED scores based on six significant explanatory variables. (*n* = 4628 Nearctic and Neotropical bird communities.)

global versus local ED corr. predicted by	*β* estimate	*p*
latitude	0.001	0.0001
species richness	0.12	0.0001
PD/species richness	−0.01	0.26
years surveyed	−0.0007	*0.003*
distance to coast	−0.00001	0.0001
coastal (habitat)	0.015	*0.02*
cropland (habitat)	0.001	0.76
flooded (habitat)	0.005	0.72
forested (habitat)	0.016	0.0001
urban/bare (habitat)	−0.01	0.35

Importantly, in spite of the moderate relationships between global and local measures of evolutionary isolation, nearly twice as much local PD was lost when removing the top 500 high scoring most evolutionary isolated species from each community compared with the removal of species at random (mean *p* < 0.03 for ED, PE and APD, *n* = 4628, multiple testing accounted for using false discovery rate control R [28]; table 2).

**Table 2.** Mean loss of biodiversity value when high global scoring species (in global top 500) are lost from each of 4628 communities. (PD value represents the (total community PD minus PD of community when all of the top 500 species are removed). Random represents the loss when an equal number of species are removed randomly. Mean pairwise distance (trait) value represents change in mean pairwise trait distance from the unaltered community to the community with all of the top 500 species removed. Positive values represent more closely related species in depleted community, and zero represents no change in the average relatedness of communities.)

metric	biodiversity measure	top 500	random	*p*
APD	PD	1080.39	446.67	>0.001
ED	PD	556.74	264.37	>0.001
PE	PD	489.99	294.87	0.03
APD	mean pairwise trait distance (TAPD)	0.032	0	>0.001
ED	mean pairwise trait distance (TAPD)	0.012	0	0.08
PE	mean pairwise distance (TAPD)	0.008	0	0.14

### (b) Trait scores

We found a weak positive correlation (*ρ* = 0.21) between ED score and TU when calculating both sets of scores globally. For the other two global measures of evolutionary isolation, APD was more strongly correlated to global TU (*ρ* = 0.43), while global PE was only weakly related to globally calculated TU (*ρ* = 0.09). Similar patterns were seen with TAPD (results not shown).

**Figure 3.**
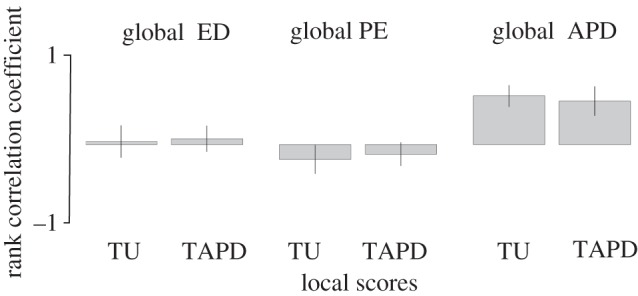
Mean correlation between three global evolutionary isolation scores (ED, PE, APD—definitions in text) and local trait minimum distance to other species in the community (local TU) and average trait pairwise distance to other members of the community (local TAPD) for 4628 bird communities across North and tropical South America.

Alternatively, if we compare the correlation of the global phylogeny-based scores (ED, PE and APD) to TU and trait pairwise difference calculated at the community level, a different relationship is revealed. At this smaller spatial scale, only one of the globally calculated evolutionary isolation metrics, APD, remains positively correlated to our community based trait measures (TAPD *ρ* = 0.42, s.d. = 0.09; TU *ρ* = 0.38 s.d. = 0.13; figure 3). Global ED scores and global PE scores were unrelated or negatively related to local TU (figure 3). Removing alien species and vagrant species from the analyses had minimal impact.

Removing the top 500 high scoring global APD species from all communities resulted in reduction in an average mean trait pairwise distance of 8%, meaning that the remaining species tended to be more similar in terms of their functional traits compared with the unperturbed community. This difference was much higher than expected from random species removal (0; *p* < 0.001; table 2). Removing the globally high scoring ED and PE species had the same effect in reducing the mean trait relatedness of the community but not significantly more than random removal (table 2).

## 4. Discussion

There appears to be potential for close agreement between different metrics used to create conservation priorities at different spatial scales for Nearctic and Neotropical birds, but this relationship varies widely among communities and depends on the precise metric used. While metrics to measure different facets of evolutionary isolation have proliferated over the past two decades, the principal metric employed in active global-scale conservation thus far has been ED [6]. However, we find that the global version of this metric had varying effects. Prioritizing evolutionary isolated species using ED safeguarded local PD on average (retaining almost twice the local tree compared with losing species on average), but the effect on prioritizing overall local trait variation showed no strong trend. The application of this global-scale metric of evolutionary isolation to community-scale conservation may therefore depend on what one wants to conserve. To date, there has been little evaluation of the relative benefits of using ED versus other metrics that are available, and also little evaluation of the properties that might matter in selecting an evolutionary isolation score for conservation [24].

Our results, however, suggest that an as-yet-unused and unproven metric of evolutionary isolation, global APD, is strongly related not only to its local community-level version but also to community-level TU. Therefore, even when setting conservation priorities for an entire clade at broad spatial scale, this metric will preferentially choose species that, within their community, have unusual sets of traits. Furthermore, APD is also the most effective metric at capturing community PD (despite performing worse than ED in global-based analyses [29]). If these community-level properties of APD are considered desirable, our results suggest APD may be a useful metric for setting conservation priorities across spatial scales, although more work is needed to confirm this.

A significant downfall of the APD metric approach is that little is known about how it relates to evolutionary processes and this represents a significant barrier to it being recommended as a conservation tool in the future. Interestingly, the only process-based evolutionary isolation metric known to the authors, character rarity [30], which attempts to model genetic information on phylogenies under different models of evolution, produces values that are almost identical to APD with its default parameter setting. By altering these settings and comparing outputs, character rarity might prove to be a useful tool to offer insights into what APD is measuring. Finally, as there is double counting of branches when calculating scores, APD may also prove to have analytically undesirable properties.

Two of the metrics examined here (ED and PE) principally or wholly are measuring the length of species' terminal branch [24]. One benefit of taking such an approach is that it is conceptually simple: this terminal branch represents all of the features that have evolved since a species split from its nearest extant relative. However and importantly, in large and incomplete phylogenies, the terminal branches are often likely to be incorrect: for instance, in the phylogenies used thus far to set conservation priorities for the Evolutionarily Distinct Globally Endangered (EDGE) programme [11,12,31], species from data-poor species groups have either been awarded all the same terminal branch or a simple evolutionary model has been used to roughly estimate branch lengths.

The third metric evaluated in this study (APD) takes into account all branches in the tree by averaging the distance along the tree from a target species to all the other species in the phylogeny. Any occasional incorrect branch lengths (particularly the terminal branch) will, therefore, provide only a limited amount of incorrect information to a final metric value. Using a metric that is not so strongly reliant on the terminal branch may also have other benefits associated with it. Most changes to taxonomic identity and phylogenetic relationships are likely to occur in localized areas of the tree, for example, through taxonomic revisions in a particular genus or family. Once a reasonable phylogeny is established for a given group [16,32], very large changes in the hypothesized relationships among species are less likely [8]. It then follows that metrics of evolutionary isolation that are less affected by tip-level phylogenetic data should be less susceptible to changes in conservation rank (e.g. species being elevated to higher categories of risk, or being down-listed as a result of concerted conservation intervention [11,33]). Interestingly, APD is commonly used to measure the overall relatedness of communities [14]. How this approach links to the distribution of species-level measures could be an interesting avenue to explore in a conservation context.

Previous work has shown support for the hypothesis that high ED scoring mammal species are moderately ecologically unusual [11,34], supporting the use of ED as a conservation metric. The moderate correlations we report here are consistent with these earlier studies. Notably, these previous studies were carried out at a clade level (i.e. comparing species within families and orders), which makes intuitive sense for a global-scale scheme of conservation priority setting for an entire taxon. However, species tend broadly to interact at the community level, where species from a number of clades may also interact. Here, we consider the situation where conservation priorities are set at the global level and show that there is no link between global ED and unusual local trait distinctiveness or complementarity. By contrast, global APD again is a fairly good surrogate for both. Furthermore, species-level APD mirrors previous work examining the functional ‘originality’ [35] of species at the community level, suggesting a potential to develop analytical links between phylogenetic and ecological conservation priorities. Certainly, if such a link can be made, then the APD metric may have some appealing properties: being able to capture aspects of ecosystem function while setting priorities at the global level using data-cheap approaches would be a valuable goal. However, this remains untested.

We observed strong spatial patterns of correlation between metrics among the communities of Nearctic and Neotropical bird species tested. The strongest correlations between global and local measures of all three metrics were observed in central continental areas, the tropics and towards northern limits of where bird communities were surveyed, and particularly for communities in the central prairie regions of the United States as well as the northern boreal forest. While we did not start out with a testable prediction of geographical patterns of the correlations of global and local values for the three metrics that we considered, we offer some observations. In the communities with strongest correlations (typically those in north and central United States), birds tend to have larger global ranges on average than those species in tropical and sub-tropical communities [36]. This may mean that, at each location, tropical communities are more likely to just sample just one or two species from the many large tropical bird clades, e.g. antbirds (*Thamnophilidae*). In addition, these communities with strong correlations tend to have more species from distinct but widespread guilds such as raptors, waterfowl and waders. By contrast, most tropical bird communities in our analysis, perhaps especially those on Christmas Bird Count routes (the design of which is not systematic), are tropical forest and woodland communities, which are almost entirely dominated by passerines and near passerines. This general difference suggests that the presence of a few species with a high isolation score (generally non-passerines) could weakly affect rank correlations. Further, previous work has also suggested areas in central North America have higher numbers of species with top 10% highest ED scores than surrounding areas [12]. An alternative driver here may be owing to some type of ecological filtering, such that some communities with a high correlation coefficient between global and local metrics of evolutionary isolation are comprised of few relatively complete clades (e.g. waders), such that tip lengths (PE scores) are similar at the two geographical scales.

Our analysis of Neotropical and Nearctic birds provides one of the first examples of how relationships between global and local measures of evolutionary isolation might be evaluated. In light of the patterns we report, we need more work, including studies uncovering the relationship between phylogenetic distance and meaningful trait distances at both global and, importantly, local levels. As the use of approaches that help prioritize at risk species that comprise disproportionately large amounts of evolutionary history continues to grow, it is important that local community-scale conservation projects can make use of the many available techniques for evaluating those species which need focused conservation attention. It is feasible that priority setting at a local scale will start to make greater use of these approaches in order to manage and integrate cost savings to projects that have very limited funding resources. However, the next step for conservation must be to determine how such metrics should be applied.
